# Assessing the Economic Burden and Benefit-cost of Treating Attention-deficit Hyperactivity Disorder in Germany

**Published:** 2013-11-12

**Authors:** Nikolaos Kotsopoulos, Mark P. Connolly, Esther Sobanski, Maarten J. Postma

**Affiliations:** 1 Unit of PharmacoEpidemiology & PharmacoEconomics, Department of Pharmacy, University of Groningen, The Netherlands; Global Market Access Solutions, St Prex, Switzerland; 2 Central Institute of Mental Health Mannheim, Department of Psychiatry & Psychotherapy, Medical Faculty Mannheim, University of Heidelberg, Mannheim, Germany; 3 Unit of PharmacoEpidemiology & PharmacoEconomics, Department of Pharmacy, University of Groningen, The Netherlands

**Keywords:** economic evaluation, economics, burden of adhd, adhd, human capital

## Abstract

**Background:** Attention-deficit hyperactivity disorder (ADHD) is a condition which has been consistently documented to impact educational outcomes. Children with ADHD are regularly found to have lower educational attainment and increased likelihood of dropping out of school compared to children without ADHD.

**Objectives:** To project the long-term societal economic consequences of reduced educational attainment, as measured by total lifetime earnings, in an untreated cohort of individuals diagnosed with ADHD in childhood, in Germany. In addition, this research aims at illustrating a cost-benefit analysis framework which could be applied to economically appraise the rate of return from investments in hypothetical health interventions targeting ADHD.

**Methods:** Observational ADHD evidence was collated with demographic and human capital economics methods to quantify ADHD’s impact on educational attainment and long-term labour outcome in Germany. The theoretical benefits deriving from effective interventions targeting ADHD were also quantified.

**Results:** It was estimated that the average per capita lifetime earning loss associated with ADHD was €92,000 suggesting a societal loss of €2.93 billion from a single cohort (n=31,864). The benefit-cost analysis suggested that reasonably effective intervention may justify considerable investment in ADHD targeted intervention.

**Conclusions:** Considering the broad economic consequences of the condition might suggest that interventions which change the life course of individuals with ADHD could offer cost-savings and influence future economic outputs.

## 1. Background

Several socio-demographic variables are deemed as potential determinants of a child's future earnings. In human capital economics, the most explored causal relationship is between earnings and quantity and/or level of education attained.[1-4] Reduced school performance has some immediate societal cost implications, but more importantly, reduced school achievement may reduce lifetime opportunities because of education-linked wage effects that will persist over the course of life.[5,6] An individual's cognitive development and school performance is known to be influenced by a number of environmental factors. Recently, a set of paediatric conditions has been identified as potentially negative influencers of performance.[7] In a review of studies assessing the impact of paediatric conditions on school performance Karande and Kulkarni[8] documented morbidity, neurobehavioral deficits and psychiatric disorders that may result in reduced school performance. Most of the identified studies assessed the effect of medical conditions on school performance and absenteeism whilst few studies assessed the resulting long-term educational attainment and the long-term labour market outcome.

In children with asthma, a study of school achievement reported that although asthmatic children performed as well as their healthy counterparts, there was evidence in labour market performance indicating that individuals with asthma during childhood experienced disadvantages in later employment.[9] A review by Milton and colleagues[10] reported that people with childhood-onset diabetes might experience disadvantages in employment, and had a lower income in adulthood, although diabetic complications appeared to be the most important determinants of social consequences in later life. In the area of infectious diseases, malaria appears to have life-long effects on cognitive development and on the education levels attained.[11,12]

A condition which has been clearly documented to impact educational outcomes is attention-deficit hyperactivity disorder (ADHD). ADHD is a chronic neurobiological disorder described as a condition in children and adults and characterised by a symptom cluster of inattention, hyperactivity and impulsivity according to Diagnostic and Statistical Manual of Mental Disorders, 4th Edition (DSM-IV) and International Classification of Disease, 10th Revision (ICD-10) code criteria.[13-15] The disorder is relatively common with estimated prevalence rates, in the United States and Germany, of 4-12% in children,[16] depending on the diagnosis criteria applied, and has been shown to be above 4% in adults.[17,18] The time to ADHD diagnosis can often be delayed with estimates suggesting up to 18 months until diagnosis in some childhood cases, and for ADHD adults, in the majority of cases no childhood diagnosis was reported.[18]

Numerous studies have described the impact that ADHD can have on educational outcomes. In addition, studies have demonstrated that different professional life courses are pursued by people with ADHD diagnosed as children and with ADHD in adulthood. Children with ADHD are regularly found to have lower educational attainment and increased likelihood of dropping out of school compared to children without ADHD.[19-21] A study of ADHD and non-ADHD individuals assessed the educational attainment for a German, matched-controlled population.[20] The study aimed at systematically assessing the profile of lifetime psychiatric comorbidity and psychosocial functioning in a German sample of adults with ADHD compared to a population-based, gender- and age-matched control group and at examining whether patients with ADHD and lifetime comorbid psychiatric diagnoses differed from patients with pure ADHD in their psychosocial functioning. The study demonstrated that ADHD individuals had lower education attained compared to non-ADHD individuals as well as a higher probability of being unemployed.[20]

Although reduced school performance in ADHD children has some immediate societal cost implications, more importantly the costs of reduced long-term educational attainment may reduce lifetime opportunities for ADHD individuals because of education-linked wage effects that will persist over the course of life.[5,6] In addition to the effect of ADHD on educational attainment and lifetime earnings, studies have also shown that ADHD may result in more costly health seeking behaviours compared to non-ADHD individuals.[22] The resulting total societal burden may be high and may thus justify interventions eliminating the effect of the condition on educational attainment.

By combining observational data with demographic methods and human capital economics it should be possible to quantify the earnings loss associated with the condition's impact on educational attainment and investigate the theoretical benefits-cost deriving from effective interventions targeting ADHD. This research aims to project the long-term societal economic consequences of reduced educational attainment, as measured by total lifetime earnings, in an untreated cohort of individuals diagnosed with ADHD in childhood compared with a control non-ADHD cohort, in Germany. In addition, this research aims at illustrating a cost-benefit analysis framework which could be applied to economically appraise the rate of return from investments in hypothetical health interventions targeting ADHD as well as other diseases that may affect school performance.

## 2. Methods and Data

We based our burden of disease estimations on the established relationship between education and lifetime earnings.[1-4,23] To estimate the economic consequences of untreated ADHD we linked education- and age-specific earnings in Germany to previously published data demonstrating differences in educational attainment for untreated ADHD individuals compared with non-ADHD individuals.[20] Moreover, we projected the earnings of untreated ADHD and non-ADHD individuals and conducted a cost-benefit analysis of hypothetical interventions of varying costs and effectiveness of interventions targeting ADHD. In order to project the future lifetime earnings of an average individual in Germany, we employed the simplest form of the Mincer model, which is widely used in human capital economics.[1] According to the Mincer model, lifetime earnings are a function of schooling years and work experience (Equation 1). It was considered that other explanatory variables of earnings such as gender, ethnicity, socio-economic background and geography were identical for both untreated ADHD and non-ADHD individuals. A study estimating the coefficients of a Mincer function for Germany was identified from the literature.[24] The study used the 1985-2001 German Socio-economic Panel study (GSOEP), a dataset consisting of individual and household micro-data. The coefficient estimates of the identified Mincer model were subsequently combined with educational attainment levels for ADHD and non-ADHD individuals. Earnings were adjusted to reflect 2010 prices.

The educational attainment data were derived from an observational data reported for Germany by Sobanski and colleagues.[20] The study reported that 8.6% of untreated ADHD individuals held a university degree and 61.4% graduated from occupational training whilst the rest completed secondary education. In contrast, the corresponding figures for non-ADHD were 25.7% and 60%, for university and occupational training, respectively. In addition, the study reported that unemployment rate was 25% higher for untreated ADHD individuals compared with the control group. The projected earnings were adjusted for unemployment using current age-specific unemployment rates in Germany accounting for 25% higher unemployment in untreated ADHD individuals as previously described.[20] Earnings were converted to 2010 at the current annual cost inflation rate of 2.80%.[25] Individuals were assumed to enter the labour force at different ages, based on their educational attainment and starting from the age of 18. The average retirement age was considered to be 65 years which was the time horizon of this economic analysis. Both costs and benefits were discounted at 1.75%.[26]

A cohort demographic model was developed simulating the lifetime of untreated ADHD and non-ADHD individuals. The model assumed identical survival probabilities for both cohorts which were, in turn, obtained from the German life tables.[27] The prevalence of untreated ADHD in Germany[18] was applied to an annual 2010 birth cohort[27] to estimate the size of the untreated ADHD and non-ADHD cohorts. By combining the results of the Mincer model with the demographic cohort models, the Present Value (PV) of total lifetime earnings were estimated for untreated ADHD and non-ADHD individuals (Equation 2). Subsequently, the difference in PV of total lifetime earnings of untreated ADHD compared to non-ADHD individuals was estimated (Equation 3). The difference between PV for ADHD and non-ADHD cohorts represented the societal burden associated with ADHD in Germany from a single birth cohort.

Cost-benefit analysis of hypothetical interventions or educational programs targeting ADHD of varying costs for a range of effects on educational attainment was explored to assess whether educational gains can justify the program costs. In this context we evaluated any hypothetical program that influences educational attainment in a matrix of costs and outcome benefits. Interventions' duration was assumed to be 11 years and the start age was set at 7 years. The total costs for a hypothetical program were summed and discounted to reflect the PV of the investment at the age of delivery (Equation 4). For each hypothetical intervention, the incremental PV of total lifetime earnings compared to untreated ADHD was derived. The latter increment represented the benefit of the intervention. Subsequently, for each intervention a cost-benefit analysis was conducted. The Net Present Value (NPV) associated with the program cost was calculated by subtracting intervention costs from benefits (Equation 5). Benefits were defined in terms of increasing the percentage attaining university degrees. In addition, a benefit-cost ratio was estimated (Equation 6).

$$\ln Y_{it} = \alpha + a_1 \text{School years}_i + a_2 t + a_3 t^2 \quad (1)$$

$$PV_{lifetime\ earnings\ i} = \sum_{1}^{65} \frac{Y_{it}\,(1-U_t)}{(1+r)^t} S_{it} \quad (2)$$

$$\text{Societal burden of ADHD} = PV_{\text{lifetime earnings without ADHD}} - PV_{\text{lifetime earnings ADHD}} \quad (3)$$

$$PV_{ADHD\ intervention\ costs} = \sum_{7}^{18} \frac{C_t}{(1+r)^t} S_{ADHD\ t} \quad (4)$$

$$NPV_{\text{intervention}} = (PV_{\text{lifetime earnings treated ADHD}} - PV_{\text{lifetime earnings untreated ADHD}}) - PV_{\text{ADHD intervention costs}}) \quad (5)$$

$$\text{Benefit-cost ratio} = (PV_{\text{lifetime earnings treated ADHD}} - PV_{\text{lifetime earnings untreated ADHD}}) : PV_{\text{ADHD intervention costs}} \quad (6)$$

| | |
|---|---|
| $i$ | ADHD status (with ADHD/without ADHD) |
| $t$ | Age (year) |
| $a_1$, $a_2$, $a_3$ | The coefficient of the Mincer function for school years, age and age$^2$, respectively |
| $S_{it}$ | Survivors at age *t* based on ADHD status (with and without ADHD) |
| $S_{ADHD\ t}$ | Survivors with ADHD at age *t* |
| $r$ | Discount rate |
| $Y_{it}$ | Earnings at age *t* based on ADHD status (with and without ADHD) |
| $U_t$ | Unemployment rate at age *t* |
| $C_t$ | ADHD intervention costs at age *t* |

### 3. Results

Evidence for the adult epidemiology of ADHD in Germany,[18] was used to estimate the presence of ADHD in an annual birth cohort of 677,947, born in 2010. Adults' epidemiology data were used since this research aims to investigate the economic impact in ADHD in adult life. Hence, it was estimated that 31,864 will be diagnosed with ADHD. The number of ADHD diagnoses defined the cohort sizes for the untreated ADHD and non-ADHD cohorts. Figure 1 shows the estimated age-specific earnings curves (in 2010 wages) for an average individual with and without ADHD. As a result of the higher probability of higher educational attainment, a non-ADHD individual is expected to have higher earnings compared to an average ADHD individual. Figure 2 illustrates the cumulative present value of earnings for the untreated and non-ADHD cohorts. The PV of total lifetime earnings for the untreated ADHD and non-ADHD cohorts were estimated at €34.03 billion and €36.96 billion, respectively. The results suggest that a single birth cohort in Germany ADHD is expected to result in a societal loss of €2.93 billion, as defined by the PV of total lifetime earnings.

**Figure 1. Estimated Age-specific Annual Earnings of an Average ADHD and Non-ADHD Individual**

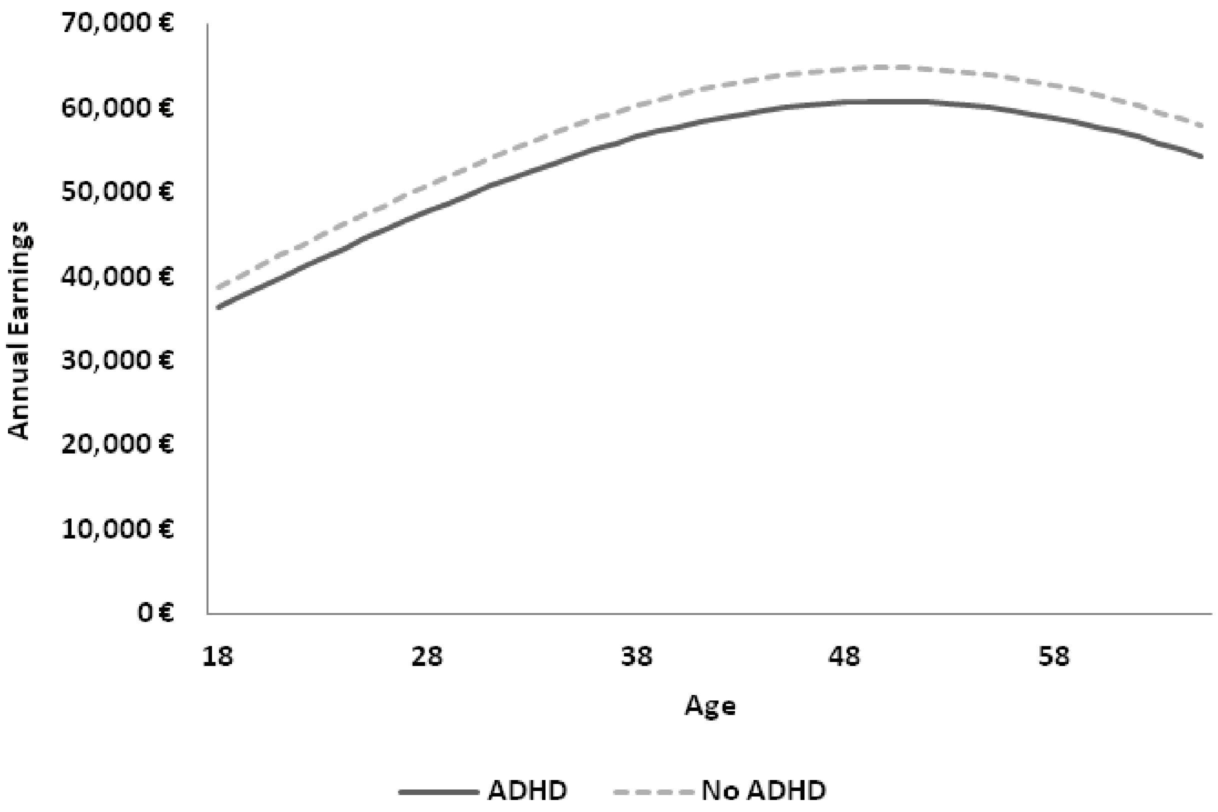

ADHD=attention-deficit hyperactivity disorder

**Figure 2. Cumulative PV of Total Annual Lifetime Earnings for a Cohort (n=31,864) of ADHD and Non-ADHD Individuals**

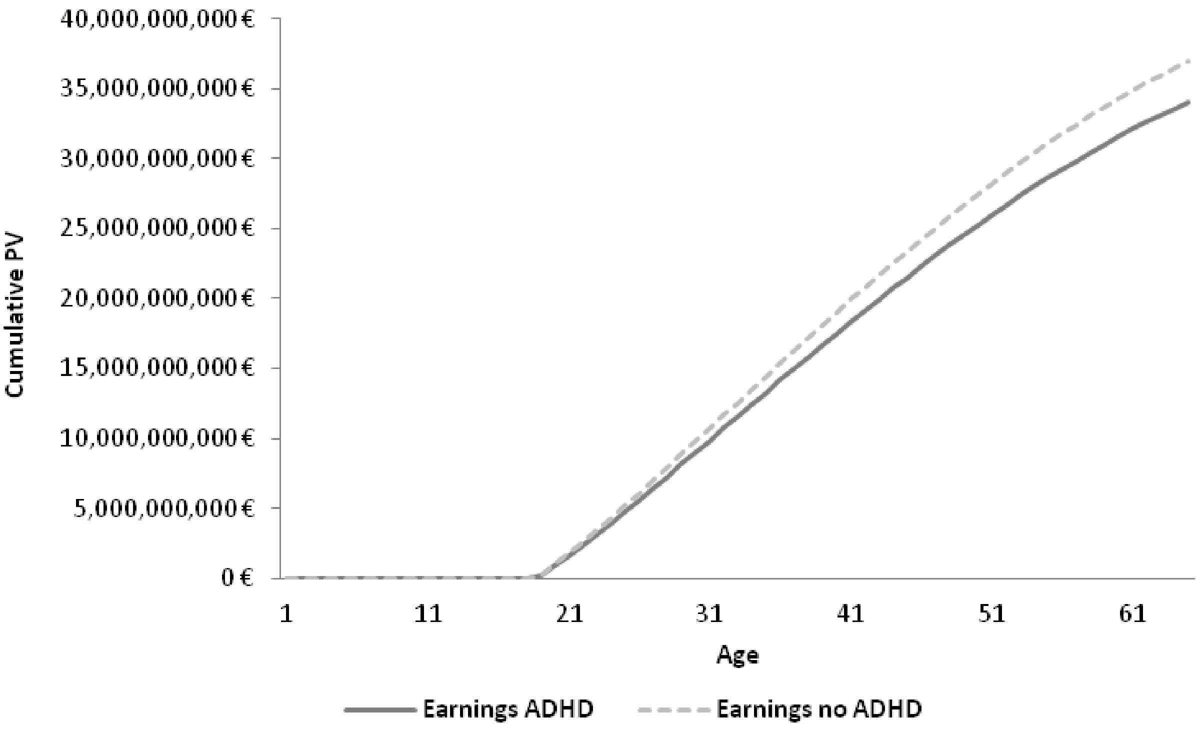

ADHD=attention-deficit hyperactivity disorder; PV=present value

In the observational study by Sobanski *et al.*[20] it was reported that although individuals with ADHD had approximately the same rate of completing occupational training, the proportion of individuals attaining a university degree was considerably higher for non-ADHD individuals (8.6% versus 25.7%). It was therefore assumed that an effective program targeting ADHD should increase the proportion of individuals accomplishing university level education. We therefore hypothesised that interventions or educational programs may benefit the educational mix of treated ADHD cohorts by increasing the proportion of individuals attaining a university degree. For different proportional increases in the number of treated ADHD individuals attaining university education and for various annual intervention costs, the NPV of investing in treating ADHD was calculated. The resulting NPVs for the different scenarios are illustrated in Table 1.

In addition, for each combination of effectiveness and annual cost the benefit–cost ratio was calculated Table 2 illustrates the resulting cost-benefit ratio. The results indicated that a small increase in university attendants may result in positive social rate of return at a reasonable cost. The cost-benefit analysis scenarios based on hypothetical education gains shows that a reasonably effective intervention (e.g. increasing university degrees from 8% to 12% in treated ADHD individuals) may justify an investment of €1,900 per year per ADHD individual and result in aggregate societal benefit.

**Table 1. Cost-benefit Analysis of Hypothetical Interventions: NPV* in Million Euros for different Annual Costs and Improvements in Educational Attainment**

| Intervention's effectiveness in increasing university attainment of ADHD individuals | Annual cost of treatment | | | | | | | | |
|---|---|---|---|---|---|---|---|---|---|
| | € 100 | € 400 | € 700 | € 1,000 | € 1,300 | € 1,600 | € 1,900 | € 2,200 | € 2,500 |
| 5% | € 16.7 | -€ 66.9 | -€ 150.5 | -€ 234.1 | -€ 262.0 | -€ 345.6 | -€ 429.2 | -€ 512.8 | -€ 596.4 |
| 10% | € 61.3 | -€ 22.3 | -€ 105.9 | -€ 189.6 | -€ 217.4 | -€ 301.0 | -€ 384.7 | -€ 468.3 | -€ 551.9 |
| 20% | € 150.2 | € 66.6 | -€ 17.0 | -€ 100.6 | -€ 128.5 | -€ 212.1 | -€ 295.7 | -€ 379.3 | -€ 462.9 |
| 30% | € 239.0 | € 155.4 | € 71.8 | -€ 11.8 | -€ 39.7 | -€ 123.3 | -€ 206.9 | -€ 290.5 | -€ 374.1 |
| 40% | € 327.6 | € 244.0 | € 160.4 | € 76.8 | € 48.9 | -€ 34.7 | -€ 118.3 | -€ 201.9 | -€ 285.5 |
| 50% | € 416.1 | € 332.4 | € 248.8 | € 165.2 | € 137.3 | € 53.7 | -€ 29.9 | -€ 113.5 | -€ 197.1 |
| 60% | € 504.3 | € 420.7 | € 337.1 | € 253.5 | € 225.6 | € 142.0 | € 58.4 | -€ 25.2 | -€ 108.9 |
| 70% | € 592.4 | € 508.7 | € 425.1 | € 341.5 | € 313.7 | € 230.0 | € 146.4 | € 62.8 | -€ 20.8 |
| 80% | € 680.2 | € 596.6 | € 513.0 | € 429.4 | € 401.5 | € 317.9 | € 234.3 | € 150.7 | € 67.1 |

*NPV = Benefit of intervention in terms of the total lifetime earnings (Treated versus untreated ADHD cohort; n = 31, 864) minus present value of intervention cost; derived from equation 5.

## 4. Discussion

Previous studies have reported that ADHD poses significant direct and indirect costs on the health service, the subjects' families and society. The study reported by Birnbaum[28] found that within a family with an ADHD child, approximately 50% of all direct and indirect costs were incurred by family members other than the ADHD subject. The study reported by Hakkaart-van Roijen *et al.*[29] reported that annual direct medical costs of ADHD were higher than those for children with other behavioural problems. Furthermore, the indirect costs from lost productivity of the parents of ADHD children were shown to be comparable to the direct health costs of treating the condition which were estimated to be €2,000 annually. Additionally, studies have demonstrated that ADHD adults have significantly higher rates of absenteeism suggesting that productivity losses are higher when compared with normal controls.[30] Analyses such as these while informative, are limited because they fail to take into consideration the impact that ADHD can have on future lifetime earnings and opportunities linked to educational attainment of persons with ADHD.[31,32] The analysis described in this paper deviates from previous analyses describing the indirect costs of ADHD because it estimates costs due to reduced lifetime earnings linked to final education level achieved.

The analysis described here focuses on the broader societal impact of ADHD in Germany and estimates the lost lifetime earnings linked to reduced educational attainment in a birth cohort of ADHD individuals.[20] The analysis reported focuses on the long-term impact of earnings losses attributed ADHD children as they reach adulthood.

**Table 2. Cost-benefit Analysis of Hypothetical Interventions: Benefit-cost Ratio**

| | | Annual cost of treatment | | | | | | | | |
|---|---|---|---|---|---|---|---|---|---|---|
| | | € 100 | € 400 | € 700 | € 1,000 | € 1,300 | € 1,600 | € 1,900 | € 2,200 | € 2,500 |
| Intervention's effectiveness in increasing university attainment of ADHD individuals | 5% | 1.60 | 0.40 | 0.23 | 0.16 | 0.15 | 0.11 | 0.09 | 0.08 | 0.07 |
| | 10% | 3.20 | 0.80 | 0.46 | 0.32 | 0.29 | 0.23 | 0.19 | 0.16 | 0.14 |
| | 20% | 6.39 | 1.60 | 0.91 | 0.64 | 0.58 | 0.46 | 0.38 | 0.32 | 0.28 |
| | 30% | 9.58 | 2.39 | 1.37 | 0.96 | 0.87 | 0.68 | 0.56 | 0.48 | 0.42 |
| | 40% | 12.76 | 3.19 | 1.82 | 1.28 | 1.16 | 0.91 | 0.75 | 0.64 | 0.55 |
| | 50% | 15.93 | 3.98 | 2.28 | 1.59 | 1.45 | 1.14 | 0.94 | 0.80 | 0.69 |
| | 60% | 19.09 | 4.77 | 2.73 | 1.91 | 1.74 | 1.36 | 1.12 | 0.95 | 0.83 |
| | 70% | 22.25 | 5.56 | 3.18 | 2.23 | 2.02 | 1.59 | 1.31 | 1.11 | 0.97 |
| | 80% | 25.41 | 6.35 | 3.63 | 2.54 | 2.31 | 1.81 | 1.49 | 1.27 | 1.10 |

*Benefit–cost ratio = Benefit of intervention in terms of the total lifetime earnings (Treated versus untreated ADHD cohort; n=31, 864) divided by the present value of intervention costs. Derived from equation 6).

The analysis described here suggests an average lifetime earning loss of €92,000 per individual and an aggregate loss of almost €3 billion. As the analysis does not take into consideration the short-term losses attributed to parental lost productivity caring for an ADHD child, the estimates provide here are likely underestimates. Although, based on previous studies reporting the productivity losses for parents of ADHD children, compared with per person costs described here, we believe that lost education linked earnings are more substantial when compared to the episodic productivity losses for parents.[29] Estimating the lifetime effect of reduced educational attainment resulting from disease, on earnings, could be factored into a traditional cost-benefit analysis. By generating measures typically used in the appraisals of public investments (i.e. NPV and rate of return), health interventions could be compared to other publically funded investments. Hence, the illustrated framework could be used in informing cross-sectorial resource allocation decisions.

There are several weaknesses to the modelling approach here that are worth considering. Namely, our analysis is based on the evidence generated by one exploratory observational study in Germany assessing the effect of ADHD on educational attainment thus, reflecting the local epidemiologic, social and economic characteristics. Furthermore, a secondary estimation of education-specific earnings was used in the current research. In addition, our analysis is based on the educational mix reported in that observational study since there is scarce cross-sectional evidence on educational attainment with ADHD as a control variable. Given the long-term horizon of this analysis, a modelling approach quantifying the economic impact of ADHD was considered as the most appropriate approach for assessing the societal consequences of the disorder. The presented analysis is based on several long-term projections of volatile economic figures which inevitably introduce the uncertainties commonly present in long-term forecasting economic and financial cost-benefit analyses.

The impact of social and health conditions on future earnings has been explored in previous economic studies. A framework defining the relationship between adolescent health conditions and education attainment was recently described by the World Health Organization Regional Office for Europe.[7] The authors of this framework also defined the relationship between health conditions in adolescents, and how reduced education influences future earning potential. To demonstrate this relationship, an analysis reported by Renna *et al.* observed that excessive drinking amongst teens increased the probability of graduating late from school, which can reduce future earnings resulting in an earnings loss of 1.5 – 1.8%.[33] In the spirit of these previous studies which defined the relationship between health conditions, education and future earnings, we sought to understand this relationship in ADHD and inform policy making in this area.

Healthcare resources are increasingly evaluated using cost-effectiveness analyses to estimate whether interventions represent good use of resources compared with comparable interventions.[34] In the analysis described here we deviate from this approach and seek to find whether investments in ADHD interventions offer some societal benefit, by evaluating intervention costs using discounted cash flow approach to derive the NPV of these investments. We demonstrate that hypothetical interventions or education programs that cost less than €700 annually and improve graduation rates by 30% offer approximately €70 million in discounted societal benefit (Table 1). It is worth noting that this approach underestimates the true benefits of an intervention because it does not take into consideration cost offsets or short-term productivity gains associated with parent work absenteeism, as well as the improved quality of life that is obtained from improved symptom management. In this analysis we provide a wide range of hypothetical program cost and education attainment scenarios so that a wide range of possibilities could be considered by policy makers and educators.

The perspective applied in the analysis described here, estimates the societal losses linked to reduced educational attainment, and the resulting wage penalty associated with reduced education. Whilst the estimates described here are based on the Mincer function which links education to future earnings, it is important to consider how these losses actually materialise in the real world. The underlying implication of the analysis described here is that individuals do not achieve their full potential. The consequences of this might be reduced productivity gains in society which influence wage and economic living standards. As people earn less, this would lead to reduced consumption which impacts industry and capital formation. While this is difficult to imagine at the individual level, when extrapolated to an annual birth cohort of ADHD individuals over generations the consequences are likely substantial.

Across many industrialised countries, a demographic transition is taking place from an agrarian-based economy with high fertility to that of industrialised and serviced based economies with low fertility and low mortality.[35] This on-going demographic transition has brought to light many challenges faced by industrialised economies as the number of future workers starts to decline.[36] In light of this transition, increased importance has been placed on initiatives to improve productivity and economic growth as the share of working aged adults starts to decline relative to those retired.[37] An analysis in Europe has stressed the importance of workers and human capital accumulation in the form of education and health for enabling workers to maximise productive capacity in an era of ageing populations.[38] Hence, within this policy imperative it is possible to position many healthcare and education programs which influence the productive capacity of economies.[39] ADHD is illustrative of the European policy imperative as a health condition that influences both the short-term productivity of parents, and the educational attainment of children which leads to long-term societal losses, in addition to the direct health costs of the condition.

Considering the broad economic consequences of the condition might suggest that interventions which change the life course of individuals with ADHD could offer both cost-savings and influence future economic outputs.

## Conflict of Interest Declaration

The work described here was conducted to fulfil doctoral requirements for Nikolaos Kotsopoulos. The work conducted by Nikolaos Kotsopoulos and Dr. Mark Connolly was supported by a grant from Shire Pharmaceuticals in Switzerland.

The other authors declare no conflicts of interest in relation to the work described here and hold no shares in the sponsoring organisation. PD Dr. E. Sobanski and Professor M.J. Postma received no financial remuneration for their scientific contributions to this work.

Esther Sobanski is a member of scientific advisory boards for Eli Lilly, Medice, Shire, and Janssen-Cilag. Shire Pharmaceuticals reviewed and edited the manuscript for scientific accuracy. Although Shire Pharmaceuticals was involved in the topic concept and fact checking of information, the content of this manuscript, the ultimate interpretation, and the decision to submit it for publication in Education Economics was made by the authors independently.
